# Experimental and molecular docking evidence for the protective role of *Monascus purpureus* red pigments against hydroxyapatite nanoparticle-induced testicular injury in male rats

**DOI:** 10.1038/s41598-026-44061-y

**Published:** 2026-04-01

**Authors:** Dina I. Sadek, Mokhtar I. Yousef, Mohamed A. M. El-Tabakh, Ahmed A. Hussein, Maher A. Kamel, Abeer El Wakil

**Affiliations:** 1 Department of Environmental Studies, Institute of Graduate Studies and Research, Alexandria University, Alexandria, Egypt; 2 Department of Zoology, Faculty of Science for Boys, Alazhar University, Cairo, Egypt; 3 Department of Biotechnology, Institute of Graduate Studies and Research, Alexandria University, Alexandria, Egypt; 4 Department of Biochemistry, Medical Research Institute, Alexandria University, Alexandria, Egypt; 5 Department of Biological and Geological Sciences, Faculty of Education, Alexandria University, Alexandria, 21526 Egypt

**Keywords:** Natural products, Autophagy, Rubropunctamine, Beclin-1, NF-κB, Biochemistry, Cell biology, Drug discovery, Physiology

## Abstract

Hydroxyapatite nanoparticles (HANPs) are increasingly utilized in biomedical and technological fields due to their biocompatibility and structural similarity to bone mineral. However, accumulating evidence indicates that prolonged exposure to HANPs may adversely affect the male reproductive system through oxidative stress, inflammation, apoptosis, and dysregulated autophagy. This study investigated the protective role of *Monascus purpureus* red pigments (RP), naturally derived bioactive compounds with antioxidant and anti-inflammatory properties, against HANP-induced testicular damage in adult male rats. Animals were orally treated with HANPs (88.3 mg/kg), RP (10, 20, or 40 mg/kg), or their combination for 50 days. Reproductive toxicity was assessed by analyzing semen quality, serum reproductive hormones, oxidative and inflammatory markers, apoptotic activity, expression of key autophagy-related genes (Beclin-1, LC3B, ULK1, and ATG9), and testicular histopathology. HANPs exposure caused significant reproductive impairment, evidenced by deteriorated semen parameters, disrupted testosterone, follicle-stimulating hormone and luteinizing hormone levels, elevated oxidative stress, increased NF-κB and caspase-3 activity, and upregulation of autophagy-related genes, accompanied by marked histopathological damage. Co-administration of RP markedly attenuated these alterations in a dose-dependent manner, with the highest dose (40 mg/kg) restoring reproductive function, redox balance, inflammatory and apoptotic status, autophagy signaling, and testicular architecture toward near-control levels. Molecular docking analysis revealed strong binding affinities of the major RP components, monascorubramine and rubropunctamine, to the autophagy-related protein LC3B, supporting a potential mechanistic role in autophagy modulation. Collectively, these findings demonstrate that *M. purpureus* RPs effectively protect against HANP-induced testicular toxicity through coordinated antioxidant, anti-inflammatory, anti-apoptotic, and autophagy-regulatory mechanisms.

## Introduction

Hydroxyapatite, a calcium phosphate compound with the formula Ca₁₀(PO₄)₆(OH)₂, is a naturally occurring bioceramic material that closely mimics the mineral component of bone and teeth. It can be synthesized via various methods, including precipitation, hydrolysis, and hydrothermal synthesis, or derived from natural sources such as fish bones, eggshells, and marine shells^1,2^. In its nanoparticulate form, hydroxyapatite nanoparticles (HANPs) have gained widespread attention in biomedical and biotechnological fields due to their unique physicochemical properties and high biocompatibility. Applications of HANPs include their use as drug carriers, implant coatings, and imaging agents^3^.

Despite their promising applications, the increasing use of HANPs has raised concerns regarding potential adverse effects on human health and the environment. HANPs have been shown to induce cytotoxic effects primarily through the generation of reactive oxygen species (ROS), leading to oxidative stress, inflammation, and genotoxicity^4–6^. Their interactions with critical biomolecules, including DNA and cytochrome enzymes, and potential accumulation in vital organs underscore the importance of careful toxicological evaluation^7^. Recent evidence indicates that HANPs can negatively affect the male reproductive system, impairing spermatogenesis, hormone secretion, mitochondrial function, and overall semen quality^8^.

Male infertility affects nearly 15% of the global population and is steadily increasing worldwide, with projections indicating a more rapid rise in the future^9^. Consequently, there is growing interest in identifying protective agents capable of mitigating reproductive toxicity, particularly those targeting oxidative damage and dysregulated autophagic signaling pathways, which are increasingly recognized as key contributor to testicular dysfunction^10,11^. In this context, naturally derived bioactive compounds with well-characterized antioxidant, anti-inflammatory, and cytoprotective activities are actively being investigated as complementary or preventive strategies to mitigate toxicant-induced reproductive damage, owing to their multi-target mechanisms of action and comparatively favorable safety profiles^12–14^.

One such promising candidate is *Monascus purpureus*, an ascomycetous fungus traditionally used in East and Southeast Asia for food fermentation. It produces a range of secondary metabolites, including a group of pigmented polyketides, namely red, orange, and yellow pigments, which possess various biological properties such as antioxidant, antimicrobial, anti-inflammatory, and cholesterol-lowering effects^15,16^. Red pigments (RP) such as rubropunctamine and monascorubramine have domonstrated notable potential in mitigating oxidative stress and inflammation, positioning them as promising candidates for protective interventions against nanoparticle-induced toxicity. In our previous laboratory’s experimental series, utilizing the same batch of animals and consistent dosing regimens, we evaluated the systemic protective effects of these pigments against the renal and hepatic toxicity induced by HANPs^17,18^. Despite these promising attributes, there remains a lack of comprehensive data regarding the protective role of *M. purpureus* RP against nanomaterial-induced reproductive toxicity. In particular, little is known about their modulatory effect on critical molecular pathways such as autophagy^19^, which plays a vital role in testicular homeostasis and spermatogenesis.

Given the established reproductive toxicity of HANPs and the bioactive potential of *M. purpureus* RPs, this study investigated their protective effects against HANP-induced testicular damage in adult male rats. The work evaluated semen quality, reproductive hormone profiles, testicular histopathology, and the expression of key autophagy-related genes, including *Beclin1*, *LC3B*, *ULK1*, and *ATG9*, to elucidate the underlying mechanisms. Complementary molecular docking analyses were conducted to explore potential interactions between the major RP constituents, monascorubramine and rubropunctamine, and autophagy-related molecular targets.

## Materials and methods

### Chemicals and materials

HANPs (white nano powder, size = 100 ± 30 nm, length = 20 ± 5 nm) were obtained from Nanotech Egypt Co. (Giza, Egypt). All chemicals were of high quality and analytical grade.

### Physicochemical characterization of HANPs and RPs

In our previous study, HANPs were characterized using high-resolution transmission electron microscopy (HR-TEM), Fourier-transform infrared spectroscopy (FTIR), and X-ray diffraction (XRD) to assess their morphology, functional groups, and crystalline structure^17^. Likewise, *Monascus* RP, extracted with ethanol from *M. purpureus* strain ATCC16436, was analyzed using UV–Visible spectroscopy, FTIR, and liquid chromatography–mass spectrometry (LC-MS). Detailed methodologies and instrumental parameters are provided in our earlier publication^17^.

### Experimental animal set up

Forty-eight adult male Wistar rats (180–200 g) were procured from the Faculty of Medicine, Alexandria University, Egypt. The animals were maintained under standard laboratory conditions: a controlled temperature of 25 ± 5 °C, relative humidity of 50–60%, and a 12-hour light/dark cycle. They had unrestricted access to a basal diet and tap water. Following a two-week acclimatization period, the rats were randomly assigned into eight equal groups (*n* = 6), each receiving a specific treatment. HANPs were administered at a dose of 88.3 mg/kg body weight, as per Nair and Jacob^20^, based on preliminary dose- optimization trials and literature evidence, demonstrating biological efficacy without inducing observable toxicity. *M. purpureus* RP was administered at three dose levels: 10 mg/kg (low dose), 20 mg/kg (intermediate dose based on Zhou et al.^21^, and 40 mg/kg (high dose), representing half, equal to, and double the reference dose, respectively. The experimental groups and their treatments were as follows: **Control group**: Received 4% dimethyl sulfoxide (DMSO), the solvent for HANPs; **RP10 group**: Received 10 mg/kg RP; **RP20 group**: Received 20 mg/kg RP; **RP40 group**: Received 40 mg/kg RP; **HANPs group**: Received 88.3 mg/kg HANPs; **RP10 + HANPs group**: Co-treated with 10 mg/kg RP and 88.3 mg/kg HANPs; **RP20 + HANPs group**: Co-treated with 20 mg/kg RP and 88.3 mg/kg HANPs; **RP40 + HANPs group**: Co-treated with 40 mg/kg RP and 88.3 mg/kg HANPs. All treatments were administered once daily via oral gavage for a duration of 50 consecutive days.

All experimental procedures were carried out in accordance with ARRIVE guidelines and the Guide for the Care and Use of Laboratory animals (International Council for Laboratory Animal Science, ICLAS) under the approval number AU14-211017-2-10 provided by the institutional animal care and use committee (IACUC) at Alexandria University, Egypt.

### Blood and organ collection

At the end of the experiment, the rats were fasted overnight and sacrificed under anesthesia using 3% isoflurane inhalation, with every effort taken to minimize discomfort^17^. Blood samples were collected via cardiac puncture into heparinized test tubes. Serum was separated by centrifugation at 860 x g for 20 min and stored at -20 °C until further analysis. Testes were carefully removed, freed from adherent tissues, rinsed in chilled 0.9% saline solution, and blotted dry with tissue paper. A portion of the tissue was immediately fixed in 10% formalin for histological examination. The remaining tissue was minced and homogenized (10%, w/v) in ice-cold phosphate buffer (0.25 M, pH 7.4) using a Potter–Elvehjem homogenizer. The homogenates were centrifuged at 10,000 x g for 20 min at 4 °C to remove cellular debris. The resulting supernatant was collected and stored at − 80 °C for subsequent biochemical analyses.

### Determination of semen characteristics

Epididymal sperm was collected for fertility assessment, including sperm motility, viability, morphological evaluation, and sperm count. Analyses were performed using an automated Computer-Assisted Semen Analysis (CASA) system (Germany), which provides objective and reproducible measurements of sperm parameters. This approach allows simultaneous evaluation of multiple sperm characteristics, minimizing observer bias^22^.

### Hormonal assays

Serum levels of follicle-stimulating hormone (FSH), luteinizing hormone (LH), and testosterone were measured using commercially available enzyme-linked immunosorbent assay (ELISA) kits according to the manufacturers’ instructions (Cusabio, USA). FSH levels were determined using the ELISA kit (Catalog No. CSB-E06869r). LH concentrations were assessed with the ELISA kit (Catalog No. CSB-E12654r). Testosterone levels were quantified using the rat Testosterone ELISA Kit (Catalog No. CSB-E05100r). All assays were performed in duplicate to ensure accuracy and reproducibility.

### Determination of inflammatory and apoptotic markers

The concentration of nuclear factor-kappa B (NF-κB) in tissue homogenates was quantified using a commercially available sandwich ELISA kit (Cat. No. BYEK3040; Chongqing Biospes Co., Ltd, China), according to the manufacturer’s protocol. The enzymatic reaction produced a color change, which was terminated by adding an acidic stop solution. Absorbance was measured at 450 nm using a microplate reader, and NF-κB concentrations were calculated based on a standard curve.

Tissue caspase-3 activity, an established marker of apoptosis, was assessed using a colorimetric Caspase-3 Activity Assay Kit (Elabscience, USA) following the manufacturer’s instructions. The release of p-nitroaniline (pNA) from the caspase-3-specific substrate was quantified by measuring absorbance at 405 nm, which is directly proportional to the enzymatic activity of caspase-3 in the sample.

### Assessment of redox markers

Total protein content in testicular tissue samples was measured using a modified Lowry method, as described by Lowry et al.^23^. This colorimetric assay is based on the formation of a blue complex between copper ions and the tyrosine and tryptophan residues of proteins under alkaline conditions. Protein concentrations were calculated from a standard curve constructed using bovine serum albumin (BSA) as the reference standard.

Lipid peroxidation in tissue samples was evaluated by measuring malondialdehyde (MDA) levels, expressed as thiobarbituric acid reactive substances (TBARS), according to the method of Draper and Hadley^24^. In this assay, the sample is heated under acidic conditions with thiobarbituric acid (TBA), resulting in the formation of a pink chromogen with maximal absorbance at 532 nm, which is directly proportional to the MDA concentration.

Reduced glutathione (GSH) and oxidized glutathione (GSSG) levels in testicular homogenates were quantified enzymatically using the method of Griffith^25^. This method allows for the determination of total glutathione content and the specific quantification of GSSG after derivatization, providing insight into the tissue redox balance.

### Expression analysis of key autophagy-related genes using quantitative real time-polymerase chain reaction (qRT-PCR)

Total RNA was isolated using RNeasy mini kit (Qiagen, Hilden, Germany) and reverse transcribed into cDNA using miScript II reverse transcriptase (Qiagen, Hilden, Germany). Real-time PCR using specific primer sets for Beclin-1 (Accession Nº NM_001034117.1; sense: TTG GCC AAT AAG ATG GGT CTG AA, antisense: TGT CAG GGA CTC CAG ATA CGA GTG), LC3B (Accession Nº NM_022867.2; sense: CAG GAT CCA TGC CGT CCC AGA AGA CC, antisense: GTC CCT TTT TGC CTT GGT AG), ULK1 (Accession Nº NM_001108341.1; sense: CAT CCG AAG GTC AGG TAG CA, antisense: GAT GGT TCC CAC TTG GGG AGA), and ATG9 (Accession Nº NM_001014218; sense: CCC ACG GGC CCT GGA GAT CA, antisense: CCG TGC TGG CGA ACG TCC AT), was performed using SYBR green (Qiagen, Hilden, Germany) and 18s rRNA (Accession Nº NR_046237.2; sense primer: GTA ACC CGT TGA ACC CCA TT; antisense primer: CAA GCT TAT GAC CCG CAC TT) as a reference gene in a LightCycler machine (Roche, Basel, Switzerland).

### Molecular docking analysis

Molecular docking is a powerful computational technique used to evaluate the energetic and geometric compatibility of a ligand within the active site of a protein. The three-dimensional (3D) structures of the investigated compounds were generated using Gaussian 09 software and subsequently converted into Protein Data Bank (PDB) format for docking analysis^26^. The crystal structure of the target LC3B receptor (PDB ID: 5WRD) was retrieved from the RCSB PDB (http://www.rcsb.org). Molecular docking simulations were carried out to assess binding affinities and interaction profiles, while validation was achieved through re-docking of co-crystallized ligand to ensure the accuracy and reliability of the computational protocol^27^.

### Histopathology examination

Tissue sections of fixed testicular tissues (10% formalin ) with a thickness of 4–6 μm were mounted on poly-L-lysine-coated slides. The sections were deparaffinized using xylene and rehydrated through a graded series of ethanol solutions. Afterward, they were stained with hematoxylin and counterstained with eosin for histological examination. Photographs were captured using an Olympus XC30 microscope (Germany) equipped with a digital camera (Olympus UC30 camera). Representative images were taken at 100x and 400x magnification from each group.

### Statistical analysis

Data were coded and analyzed using SPSS version 22.0. The assumptions of parametric tests were verified, and the normality of continuous variables was assessed using the Shapiro–Wilk and Kolmogorov–Smirnov tests. Probability and percentage data were normalized using the arcsine square root transformation. Results were expressed as mean ± standard deviation (SD). Each experimental group included at least six replicates. Post hoc comparisons were performed using Tukey’s test following one-way ANOVA.

## Results

### Semen characteristics under different treatment protocols

**Fig. 1:**
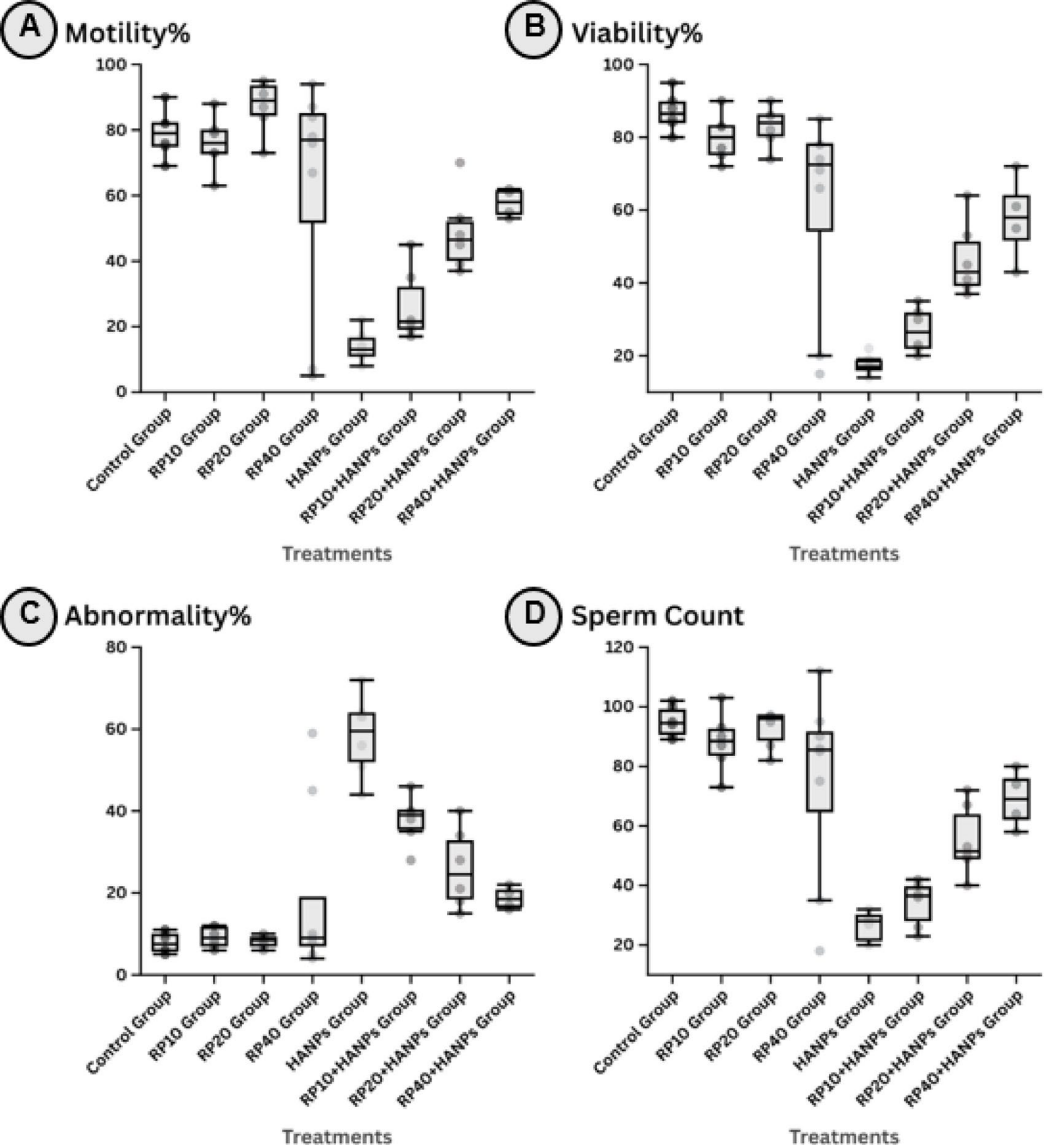
Box-plot representing semen characteristics in testicular tissues of the different studied groups. (**A**) Motility, (**B**) Viability, (**C**) Abnormality, and (**D**) Sperm count. HANPs: hydroxyapatite nanoparticles; RP: red pigment of *Monascus purpureus*. Data are presented as mean ± SD (*n* = 6).

The effects of *M. purpureus* RP and HANPs on semen parameters are presented in Table 1; Fig. 1A-D. Rats treated with RP alone at doses of 10, 20, or 40 mg/kg showed no statistically significant changes in sperm motility, viability, sperm count, or abnormal morphology compared with the control group. In contrast, exposure to HANPs (88.3 mg/kg) led to a marked deterioration in all semen parameters. Specifically, sperm motility dropped significantly to 14.00 ± 4.94%, viability to 17.50 ± 2.74%, and sperm count to 26.33 ± 5.16 per 10⁶/mL, while the percentage of abnormal spermatozoa rose sharply to 58.33 ± 10.05%, compared to 79.00 ± 7.27%, 87.00 ± 5.22%, 95.00 ± 5.22 per 10⁶/mL, and 7.83 ± 2.48% respectively in the control group.

**Table 1:** Mean ± SD of semen characteristics in testicular tissues of the different studied groups and their statistical significance.

Treatments	Motility%	Viability%	Abnormality%	Sperm Count (×10⁶/mL)
Control group	79.00 ± 7.27^ab^	87.00 ± 5.22^a^	7.83 ± 2.48^c^	95.00 ± 5.22^a^
RP10 group	76.00 ± 8.44^ab^	80.00 ± 6.57^ab^	9.17 ± 2.56^c^	88.17 ± 10.05^a^
RP20 group	87.33 ± 8.17^a^	83.00 ± 5.62^ab^	8.17 ± 1.47^c^	92.50 ± 6.44^a^
RP40 group	62.25 ± 35.62^ab^	60.88 ± 27.37^bc^	18.63 ± 21.04^c^	74.50 ± 31.76^ab^
HANPs group	14.00 ± 4.94^d^	17.50 ± 2.74^e^	58.33 ± 10.05^a^	26.33 ± 5.16^d^
RP10 + HANPs group	26.50 ± 11.06^cd^	27.00 ± 6.13^de^	37.83 ± 6.01^b^	34.00 ± 7.72^cd^
RP20 + HANPs group	48.67 ± 11.98^bc^	46.50 ± 10.27^cd^	26.00 ± 9.74^bc^	55.17 ± 12.02^bc^
RP40 + HANPs group	57.75 ± 4.43^bc^	57.75 ± 12.09^bc^	18.75 ± 2.75^bc^	69.00 ± 9.87^ab^

Means that do not share a letter are significantly different (*n* = 6).

Co-administration of RP with HANPs resulted in a dose-dependent improvement in all semen parameters. At the lowest RP dose (10 mg/kg), sperm motility and count increased to 26.50 ± 11.06% and 34.00 ± 7.72 per 10⁶/mL, respectively, while abnormalities decreased to 37.83 ± 6.01%. The intermediate RP dose (20 mg/kg) further improved motility (48.67 ± 11.98%) and sperm count (55.17 ± 12.02 per 10⁶/mL) with a concurrent decline in abnormal forms (26.00 ± 9.74%). The highest dose (40 mg/kg) showed the most pronounced protective effect, significantly restoring sperm motility (57.75 ± 4.43%), viability (57.75 ± 12.09%), and count (69.00 ± 9.87 per 10⁶/mL), along with a marked reduction in abnormalities (18.75 ± 2.75%) compared with the HANPs-only group. These findings highlight the potential of RP to mitigate HANP-induced reproductive toxicity in a dose-dependent manner.

### Evaluation of reproductive hormones across various treatment protocols

**Fig. 2:**
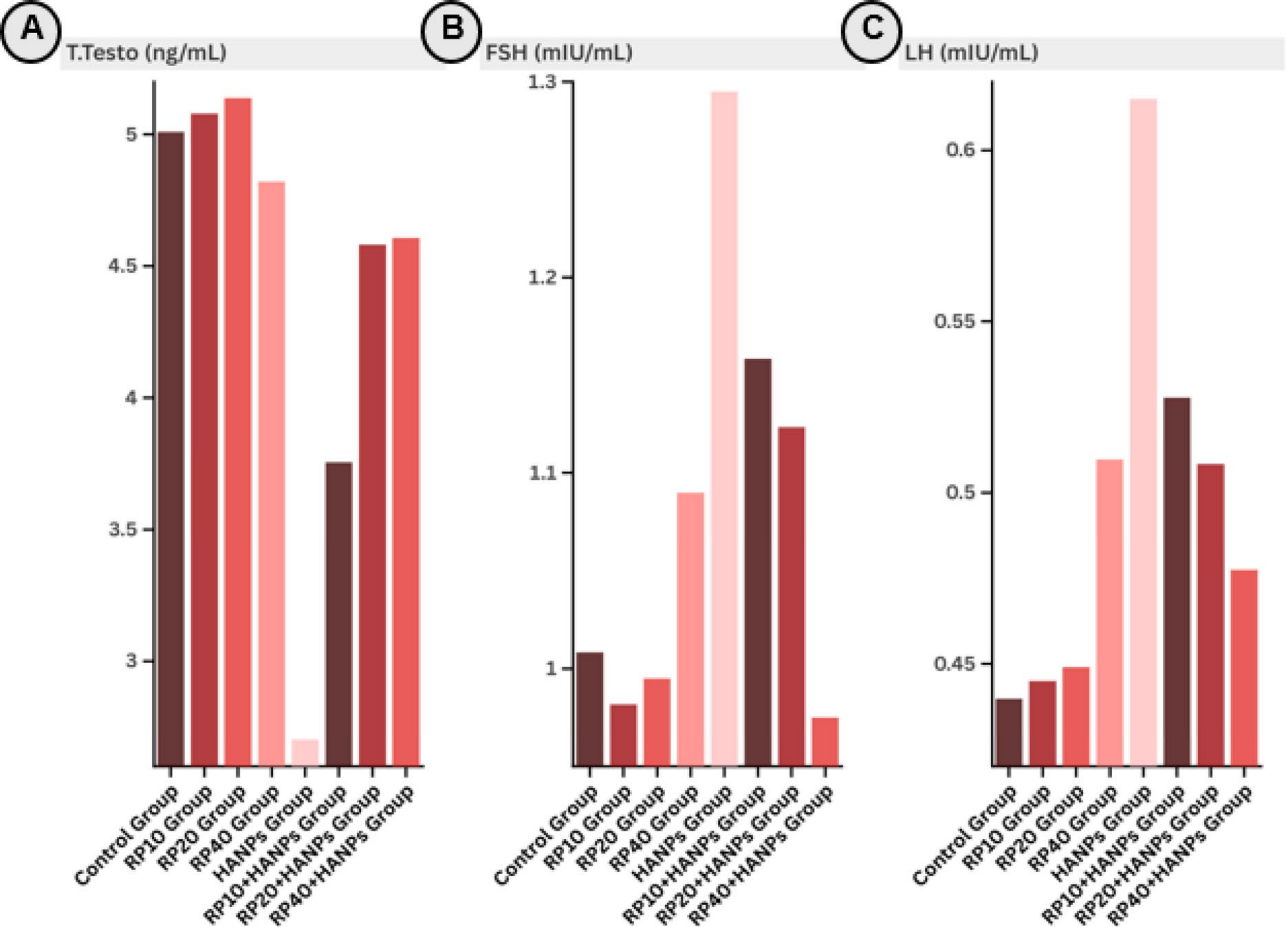
Column chart showing reproductive hormone levels in the serum of the different studied groups: (**A**) testosterone, (**B**) follicle-stimulating hormone (FSH), and (**C**) luteinizing hormone (LH). HANPs: hydroxyapatite nanoparticles; RP: red pigment of *Monascus purpureus*.

The effects of RP and HANPs on reproductive hormones are illustrated in Fig. 2A-C; Table 2. Administration of RP alone at doses of 10, 20, or 40 mg/kg did not significantly alter serum testosterone, FSH, or LH levels compared to the control group. However, exposure to HANPs resulted in a pronounced hormonal disturbance, characterized by a significant decline in testosterone levels (2.70 ± 0.64 ng/mL vs. 5.01 ± 0.55 ng/mL in control), accompanied by elevated FSH and LH levels (1.30 ± 0.10 and 0.62 ± 0.05 mIU/mL vs. 1.01 ± 0.16 and 0.44 ± 0.06 mIU/mL in control, respectively).

**Table 2:** Serum levels of reproductive hormones in the different studied groups.

Treatments	Total Testosterone (ng/mL)	FSH (mIU/mL)	LH (mIU/mL)
Control group	5.01 ± 0.55^ab^	1.01 ± 0.16^b^	0.44 ± 0.06^b^
RP10 group	5.08 ± 0.32^a^	0.98 ± 0.18^b^	0.45 ± 0.03^b^
RP20 group	5.14 ± 0.28^a^	1.00 ± 0.10^b^	0.45 ± 0.03^b^
RP40 group	4.82 ± 1.37^ab^	1.09 ± 0.17^ab^	0.51 ± 0.08^b^
HANPs group	2.70 ± 0.64^c^	1.30 ± 0.10^a^	0.62 ± 0.05^a^
RP10 + HANPs group	3.76 ± 0.58^bc^	1.16 ± 0.09^ab^	0.53 ± 0.04^ab^
RP20 + HANPs group	4.58 ± 0.38^ab^	1.12 ± 0.09^ab^	0.51 ± 0.05^b^
RP40 + HANPs group	4.61 ± 0.42^ab^	0.98 ± 0.09^b^	0.48 ± 0.04^b^

Data are expressed as mean ± SD (n = 6). Means that do not share a letter are significantly different.

Co-supplementation with RP led to a dose-dependent restoration of hormonal balance. At the highest RP dose (40 mg/kg), testosterone levels improved substantially (4.61 ± 0.42 ng/mL), while FSH and LH levels declined to near-control values (0.98 ± 0.09 and 0.48 ± 0.04 mIU/mL, respectively), indicating a strong protective effect. These results suggest that RP effectively mitigates HANP-induced reproductive endocrine disruption in a dose-dependent manner.

### Assessment of testicular inflammatory and apoptotic markers across various treatment protocols

**Fig. 3:**
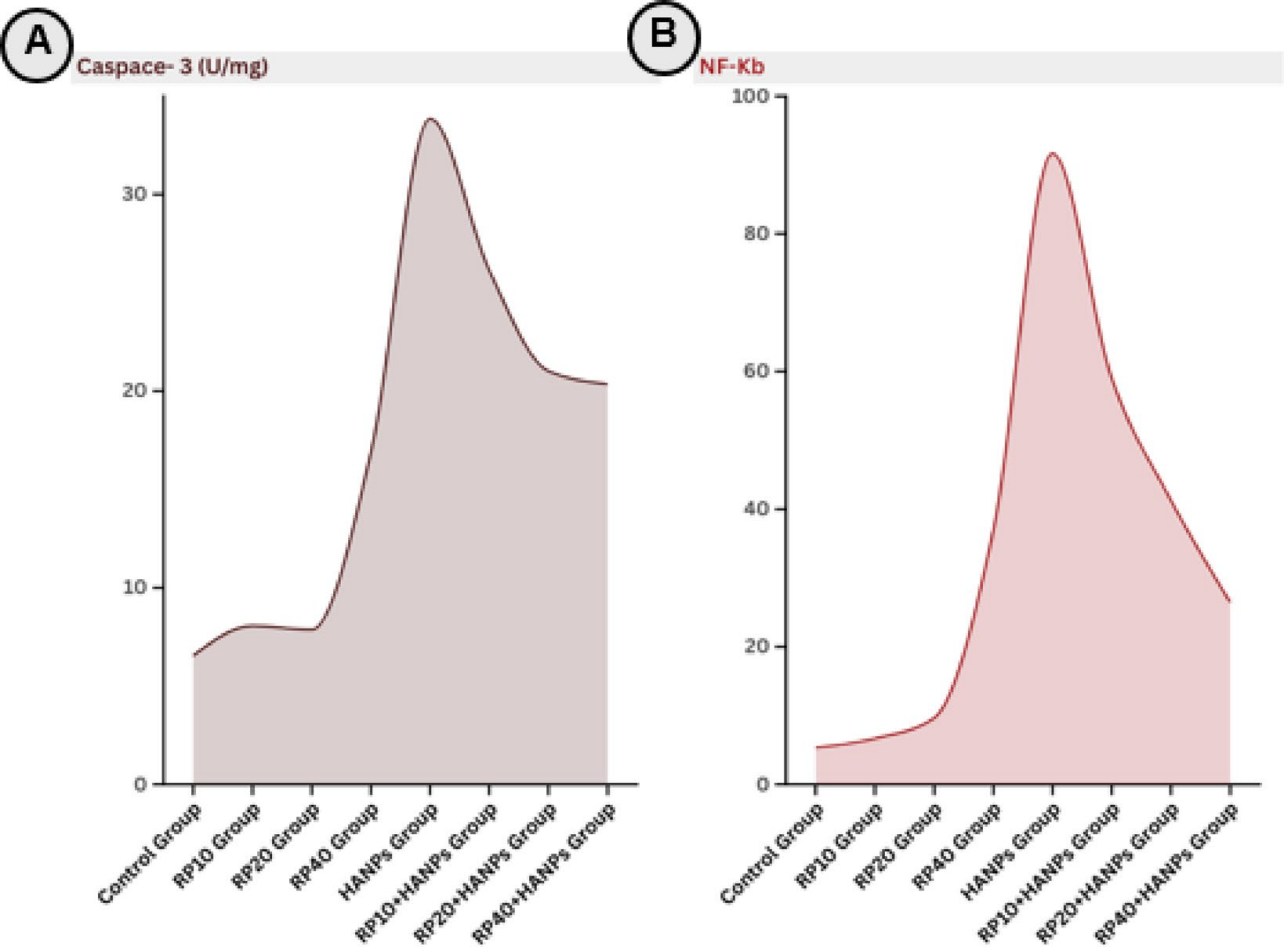
Area chart representing apoptotic and inflammatory markers in testicular tissues of the different studied groups: (**A**) caspase-3 activity and (**B**) nuclear factor-kappa B (NF-κB) content. HANPs: hydroxyapatite nanoparticles; RP: red pigment of *Monascus purpureus*.

As shown in Table 3; Fig. 3A and B, supplementation with different doses of RP alone (10, 20, and 40 mg/kg) did not cause significant changes in caspase-3 or NF-κB levels compared with the control group. In contrast, exposure to HANPs markedly elevated both caspase-3 (33.85 ± 3.05 U/mg) and NF-κB (91.64 ± 26.32 pg/mg) compared with controls (6.55 ± 1.31 U/mg and 5.35 ± 0.58 pg/mg, respectively). Co-supplementation of RP with HANPs significantly reduced these elevations in a dose-dependent manner. The most pronounced attenuation was observed with the highest RP dose, which lowered caspase-3 to 20.35 ± 3.04 U/mg and NF-κB to 26.47 ± 4.49 pg/mg, although these values remained notably higher than the normal control levels.

**Table 3:** Apoptotic and inflammatory markers in testicular tissues of the different studied groups.

Treatments	Caspace- 3 (U/mg)	NF-kb (pg/mg)
Control group	6.55 ± 1.31^d^	5.35 ± 0.58^c^
RP10 group	8.06 ± 1.00^cd^	6.66 ± 1.46^c^
RP20 group	7.87 ± 0.93^cd^	9.64 ± 2.30^c^
RP40 group	16.85 ± 13.43^bcd^	36.88 ± 44.16^bc^
HANPs group	33.85 ± 3.05^a^	91.64 ± 26.32^a^
RP10 + HANPs group	26.14 ± 4.03^ab^	59.01 ± 13.61^ab^
RP20 + HANPs group	21.01 ± 2.85^b^	41.28 ± 13.36^bc^
RP40 + HANPs group	20.35 ± 3.04^bc^	26.47 ± 4.49^bc^

Data are expressed as mean ± SD (n = 6). Means that do not share a letter are significantly different.

### Assessment of redox markers in the different experimental animal groups

**Fig. 4:**
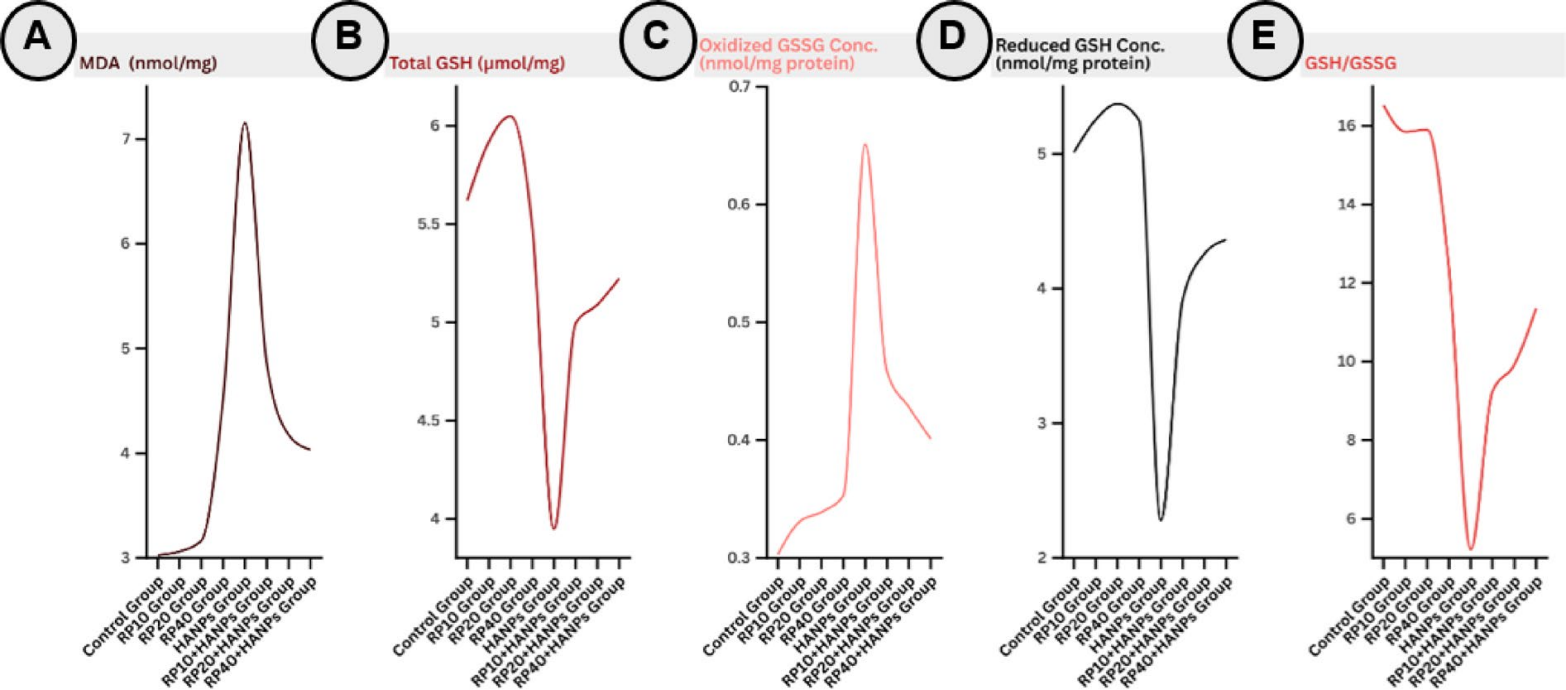
Line chart representing redox status in testicular tissues of the different studied groups: (**A**) malondialdehyde (MDA) content, (**B**) total glutathione (GSH) content, (**C**) oxidized glutathione (GSSG) content, (**D**) reduced glutathione content, and (**E**) GSH/GSSG ratio. HANPs: hydroxyapatite nanoparticles; RP: red pigment of *Monascus purpureus*.

As presented in Table 4; Fig. 4A-F, RP supplementation alone at any tested dose did not significantly alter redox parameters relative to controls. HANPs exposure significantly increased MDA levels (7.15 ± 1.39 nmol/mg tissue vs. 3.03 ± 0.23 nmol/mg tissue in controls) and oxidized GSSG (0.65 ± 0.04 nmol/mg protein vs. 0.30 ± 0.02 nmol/mg protein), while markedly decreasing total GSH (3.59 ± 0.28 nmol/mg protein vs. 5.62 ± 0.42 nmol/mg protein), reduced GSH (2.28 ± 0.34 nmol/mg protein vs. 5.01 ± 0.48 nmol/mg protein), and the GSH/GSSG ratio (3.52 ± 0.62 vs. 16.53 ± 0.95 in controls). Co-supplementation of RP with HANPs significantly and dose-dependently restored antioxidant capacity, evidenced by higher total GSH, reduced GSH, and GSH/GSSG ratio, along with decreased MDA and oxidized GSSG compared with HANPs alone. The highest RP dose produced the greatest improvement, with MDA reduced to 4.00 ± 0.15 nmol/g tissue and GSH/GSSG ratio increased to 10.92 ± 1.11.

**Table 4:** Mean ± SD of redox status parameters in testicular tissues of the different studied groups.

Treatments	MDA (nmol/mg)	Total GSH (µmol/mg)	Oxidized GSSG Conc. (nmol/mg)	Reduced GSH Conc. (nmol/mg)	GSH/GSSG
Control group	3.03 ± 0.23^b^	5.62 ± 0.49^a^	0.30 ± 0.02^c^	5.01 ± 0.48^a^	16.53 ± 1.08^a^
RP10 group	3.06 ± 0.38^b^	5.92 ± 0.44^a^	0.33 ± 0.02^cd^	5.26 ± 0.44^a^	15.85 ± 1.05^a^
RP20 group	3.17 ± 0.23^b^	6.05 ± 0.45^a^	0.34 ± 0.02^cd^	5.37 ± 0.40^a^	15.90 ± 1.18^a^
RP40 group	4.52 ± 2.28^b^	5.48 ± 1.26^a^	0.35 ± 0.02^c^	5.43 ± 0.39^a^	12.38 ± 5.65^ab^
HANPs group	7.15 ± 1.39^a^	3.95 ± 0.59^b^	0.65 ± 0.04^d^	2.28 ± 0.34^c^	5.22 ± 2.59^c^
RP10 + HANPs group	4.86 ± 0.57^b^	5.00 ± 0.35^ab^	0.46 ± 0.04^d^	3.92 ± 0.36^b^	9.23 ± 1.46^bc^
RP20 + HANPs group	4.18 ± 0.28^b^	5.09 ± 0.32^ab^	0.43 ± 0.02^dc^	4.26 ± 0.34^b^	9.92 ± 1.12^bc^
RP40 + HANPs group	4.03 ± 0.16^b^	5.22 ± 0.34^ab^	0.40 ± 0.03^c^	4.36 ± 0.28^b^	11.36 ± 1.39^ab^

Means that do not share a letter are significantly different (n = 6).

### Assessment of autophagy-related gene expression (*Beclin-1*, *LC3B*, *ULK1*, *ATG9*) across various treatment protocols

**Fig. 5:**
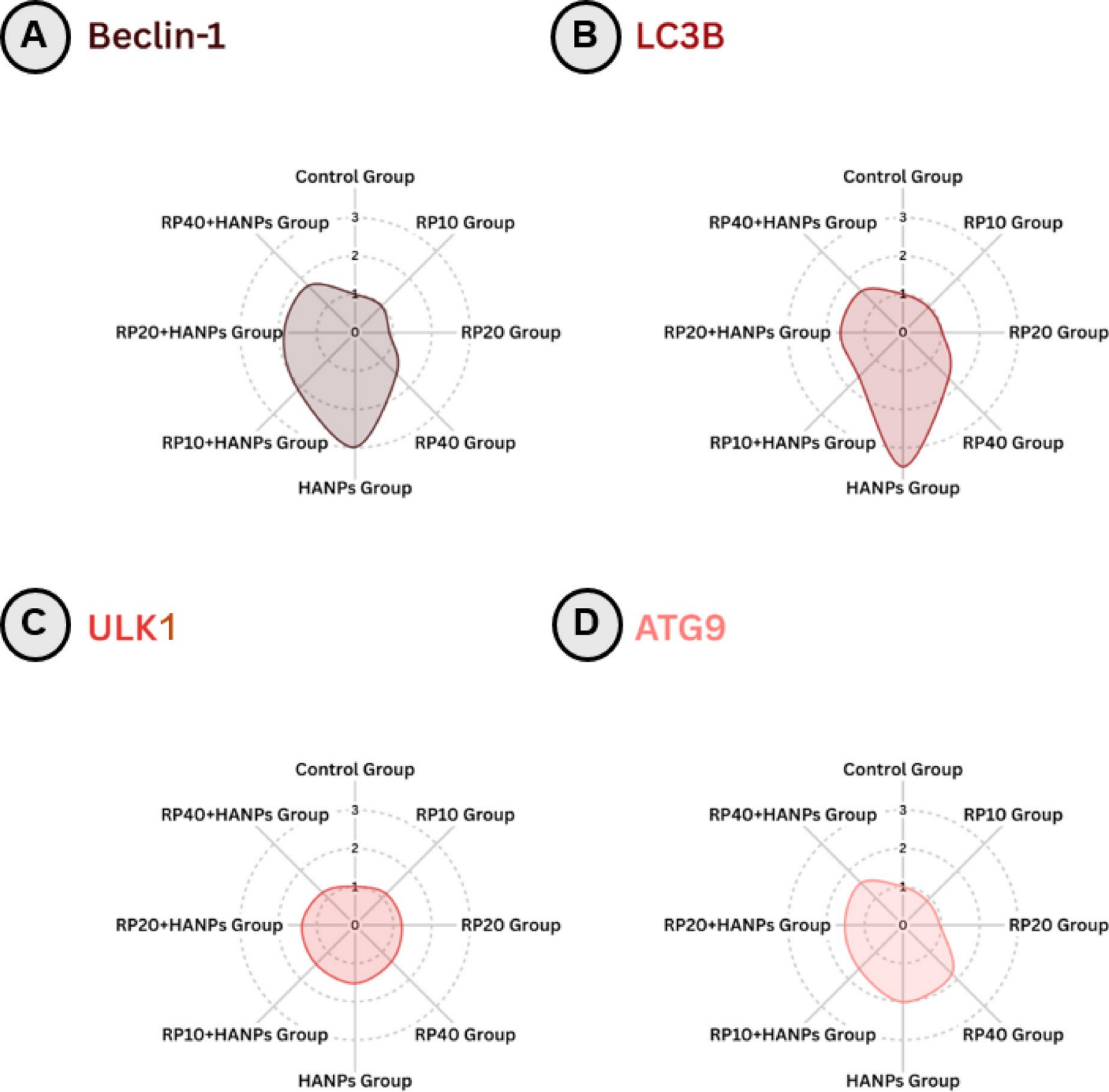
Radar chart showing autophagy-related gene expression in testicular tissues of the different studied groups under various treatment protocols: (**A**) Beclin-1, (**B**) LC3B, (**C**) ULK1, and (**D**) ATG9. HANPs: hydroxyapatite nanoparticles; RP: red pigment of *Monascus purpureus*; LC3B: microtubule-associated protein light chain 3; ULK1: Unc-51-like autophagy-activating kinase 1; ATG9: autophagy-related protein 9.

As illustrated in Fig. 5A-D; Table 5, RP supplementation alone did not significantly alter the expression of *Beclin-1*, *LC3B*, *ULK1*, or *ATG9* compared with controls. HANPs exposure markedly upregulated these autophagy-related genes (*Beclin-1*: 2.99 ± 0.41; *LC3B*: 3.50 ± 1.33; *ULK1*: 1.52 ± 0.13; *ATG9*: 2.01 ± 0.55 fold change) relative to control values. Co-supplementation with RP significantly downregulated their expression in a dose-dependent manner compared with HANPs alone. The most substantial normalization was achieved with 40 mg/kg RP, reducing *Beclin-1* to 1.73 ± 0.04, *LC3B* to 1.53 ± 0.08, *ULK1* to 1.20 ± 0.17, and *ATG9* to 1.55 ± 0.27 fold change, closely approaching control levels.

**Table 5:** Mean ± SD of autophagy-related gene expression in testicular tissues of the different studied groups.

Treatments	Beclin-1	LC3B	ULK1	ATG9
Control group	1.00 ± 0.05^c^	1.00 ± 0.24^b^	1.00 ± 0.24^b^	1.00 ± 0.25^bc^
RP10 group	0.98 ± 0.09^c^	0.94 ± 0.18^b^	1.15 ± 0.20^ab^	0.89 ± 0.14^c^
RP20 group	0.90 ± 0.08^c^	1.03 ± 0.31^b^	1.22 ± 0.14^ab^	0.97 ± 0.10^bc^
RP40 group	1.53 ± 1.19^bc^	1.68 ± 1.11^b^	1.35 ± 0.23^ab^	1.80 ± 0.61^a^
HANPs group	2.99 ± 0.41^a^	3.50 ± 1.33^a^	1.52 ± 0.13^a^	2.01 ± 0.55^a^
RP10 + HANPs group	2.10 ± 0.26^ab^	1.63 ± 0.23^b^	1.44 ± 0.20^a^	1.62 ± 0.10^ab^
RP20 + HANPs group	1.85 ± 0.15^bc^	1.63 ± 0.21^b^	1.39 ± 0.26^ab^	1.52 ± 0.20^ab^
RP40 + HANPs group	1.73 ± 0.04^bc^	1.53 ± 0.08^b^	1.20 ± 0.17^ab^	1.55 ± 0.27^abc^

Means that do not share a letter are significantly different (n = 6).

### Docking interaction against the active site of the target protein, *LC3B* (PDB ID: 5WRD)

**Fig. 6:**
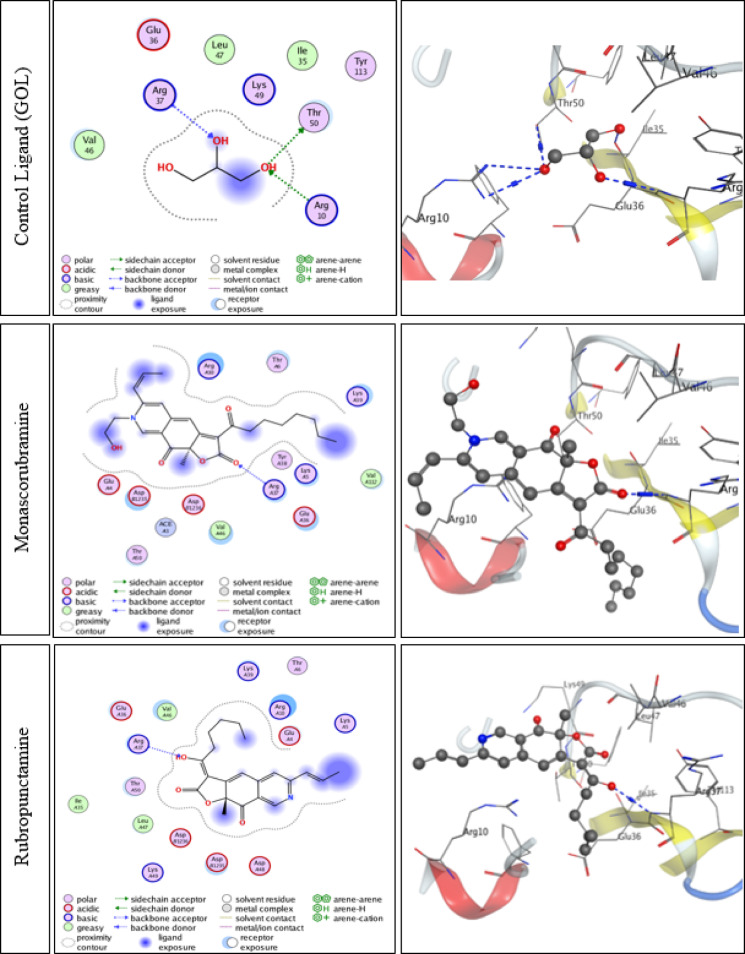
Two- and three-dimensional molecular docking representations showing the binding interactions of the co-crystallized control ligand (GOL), monascorubramine, and rubropunctamine within the active site of the LC3B receptor (PDB ID: 5WRD).

Molecular docking studies were performed to evaluate the binding interactions of monascorubramine and rubropunctamine with the LC3B receptor (PDB ID: 5WRD), using Glycerol (GOL) as the control ligand (Table 6; Fig. 6). The control ligand, GOL, displayed moderate interaction within the LC3B active site, forming hydrogen donor and acceptor bonds with residues THR50, ARG37, and ARG10, with bond lengths ranging from 2.99 to 3.30 Å and an overall docking energy score of − 4.0867 kcal/mol.

**Table 6:** Molecular docking interaction data of the co-crystallized control ligand (GOL), monascorubramine, and rubropunctamine with the active site of the LC3B receptor (PDB ID: 5WRD).

Compound	Ligand	Amino acids	Interaction	Affinity bond length (in A^o^ from main residue)	Affinity bond strength (Kcal/mol)	Energy score (Kcal/mol)
Control ligand (GOL)	O13	THR50	H-donor	2.99	-1	-4.0867
	O8	ARG37	H-acceptor	3.18	-1.6	
	O13	ARG10	H-acceptor	3.3	-0.8	
Monascorubramine	O17	ARG37	H-acceptor	2.95	-3.4	-6.8395
Rubropunctamine	O48	ARG37	H-acceptor	3.11	-0.7	-6.6515

Monascorubramine exhibited the highest binding affinity among the tested compounds, characterized by a strong hydrogen acceptor bond with ARG37 at a bond length of approximately 2.95 Å and an affinity bond strength of − 3.4 kcal/mol, resulting in a docking energy score of − 6.8395 kcal/mol. Rubropunctamine also demonstrated a stable interaction through a hydrogen acceptor bond with ARG37 (3.11 Å, − 0.7 kcal/mol), achieving a docking score of − 6.6515 kcal/mol.

The binding affinities of monascorubramine and rubropunctamine were markedly higher than that of the control ligand, indicating stronger and more stable interactions with the LC3B active site. The ranking of binding energies followed the order: monascorubramine > rubropunctamine > GOL, suggesting that monascorubramine in particular may form a more energetically favorable complex with LC3B.

### Effects of variable doses of RP, alone or in combination with HANPs, on testicular histopathology

**Fig. 7:**
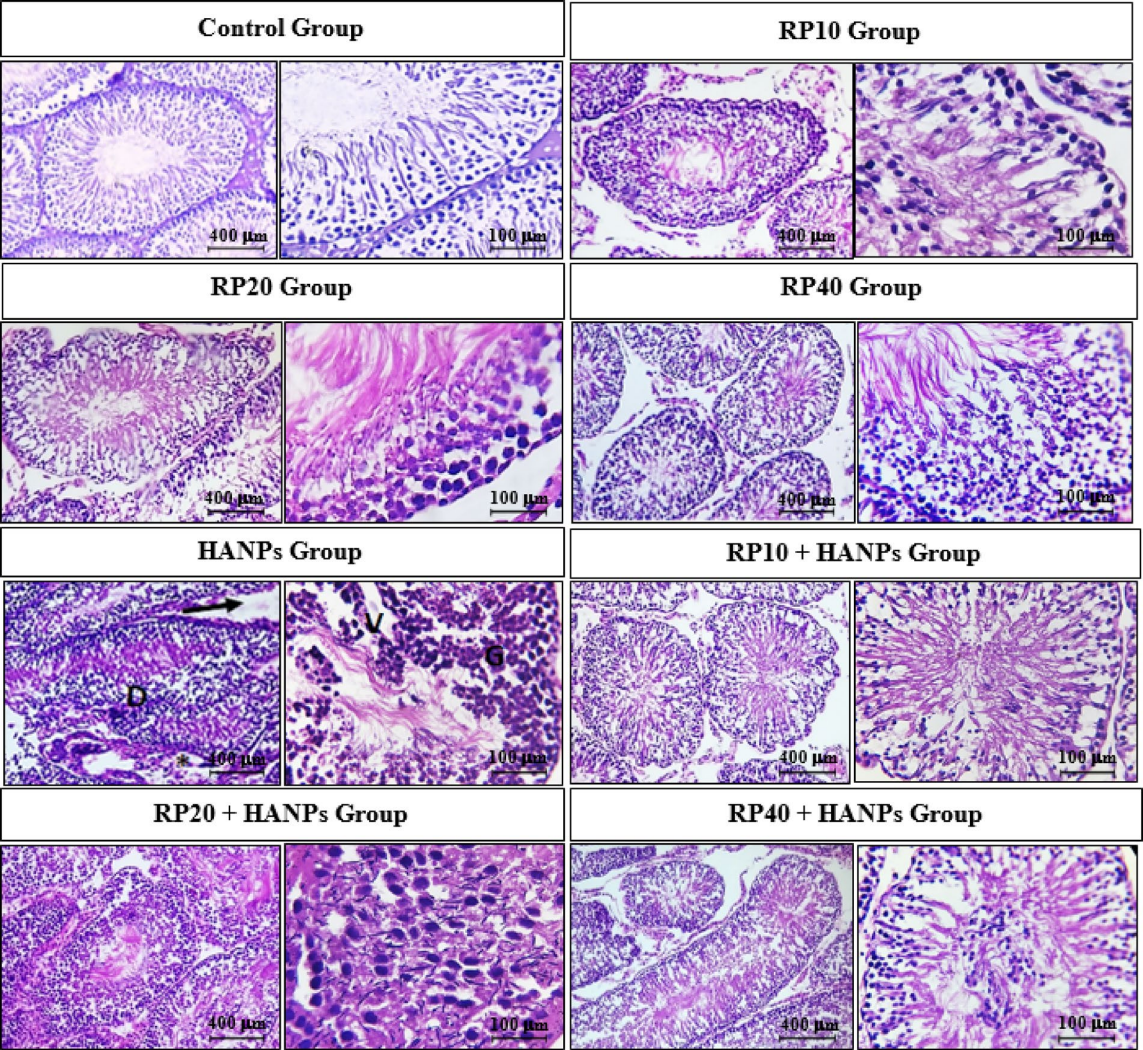
Representative photomicrographs of testicular tissue sections stained with H&E from the different experimental groups. The control and *Monascus purpureus* red pigment (RP; 10, 20, and 40 mg/kg) groups show normal seminiferous tubules and intact interstitial tissue with well-organized testicular epithelium and complete spermatogenic stages. In contrast, the HANPs group exhibits marked degeneration (D) and vacuolation (V) of seminiferous tubules, atrophy of interstitial tissue (black arrow), irregular epithelial lining (*), and disrupted spermatogenic series (G). Co-supplementation of RP with HANPs demonstrates a dose-dependent protective effect: the 10 mg/kg dose shows mild improvement with reduced degeneration and interstitial atrophy, the 20 mg/kg dose reveals more regular seminiferous tubules and improved epithelial integrity, while the 40 mg/kg dose largely restores normal testicular architecture with intact epithelium, healthy interstitial tissue, and complete spermatogenesis. Scale bars represent 400 μm at 100× magnification and 100 μm at 400× magnification.

Histological assessment of the testicular sections revealed that both the control group and rats supplemented with *M. purpureus* RP alone at all tested doses (10, 20, and 40 mg/kg) exhibited normal seminiferous tubules and well-preserved interstitial tissue (Fig. 7). The testicular epithelium were intact, displaying complete spermatogenic series with clearly distinguishable spermatogerm stages, and no structural abnormalities were observed.

In contrast, the HANPs group showed marked histopathological alterations, including degeneration and vacuolation of the seminiferous tubules, atrophy of the interstitial tissue, and irregular integrity of the testicular epithelium with disrupted spermatogerm stages.

Co-supplementation of RP with HANPs revealed a dose-dependent amelioration of these histological changes. At the lowest RP dose, seminiferous tubules appeared irregular, with only slight improvement in degeneration and reduced interstitial tissue atrophy, alongside partial restoration of the epithelial architecture and spermatogerm stages. The intermediate RP dose showed more regular seminiferous tubules, further reduction of degenerative changes, and restoration of interstitial tissue and epithelial integrity. Notably, the highest RP dose preserved normal seminiferous tubule architecture with no visible degeneration, maintained healthy interstitial tissue, and restored a fully intact testicular epithelium with complete spermatogenic activity.

## Discussion

HANPs are widely recognized for their biomedical applications, including drug delivery, gene therapy, and molecular imaging^28–30^. Despite their benefits, accumulating evidence highlights their potential toxicity, particularly to the male reproductive system, due to their small size, high surface area, and ability to generate reactive oxygen species (ROS). In the present study, long-term oral exposure to HANPs induced marked reproductive toxicity in male rats, as evidenced by histological damage, impaired semen quality, hormonal imbalance, oxidative stress, inflammation, apoptosis, and altered autophagy-related gene expression. Collectively, these findings indicate the involvement of multiple interconnected pathways rather than a single dominant mechanism.

Histopathological examination revealed that HANPs exposure severely disrupted testicular architecture, with degeneration and vacuolation of seminiferous tubules, atrophy of interstitial tissue, and irregular epithelial lining with incomplete spermatogenic stages. These alterations were consistent with significant declines in sperm count, motility, and viability, accompanied by increased abnormal sperm forms. Such structural and functional impairments are likely interlinked, as normal seminiferous tubule architecture and Sertoli–Leydig cell integrity are crucial for spermatogenesis. These findings agree with previous reports that HANPs impair Leydig cell function, disrupt spermatogenesis, and reduce semen quality^8^.

The present study also demonstrated that HANP-induced testicular damage was strongly associated with oxidative stress. HANPs significantly increased MDA levels, reflecting enhanced lipid peroxidation, while depleting GSH and lowering the GSH/GSSG ratio. This oxidative imbalance compromises sperm membrane integrity, given the high content of polyunsaturated fatty acids in sperm membranes, and contributes to reduced motility, abnormal morphology, and cell death^31^. Moreover, oxidative injury to Leydig cells disrupts testosterone synthesis^32,33^, which in turn disturbs the hypothalamic–pituitary–gonadal axis, explaining the observed testosterone decline and compensatory increases in FSH and LH levels.

Inflammation appeared to be another key mechanism of HANP toxicity. Elevated NF-κB levels in the testes indicated activation of inflammatory signaling, which can impair the blood–testis barrier, disturb Sertoli cell function, and negatively affect spermatogenesis^34–36^. Parallel to inflammation, HANPs activated the apoptotic cascade, as shown by the marked increase in caspase-3 activity. While these data support activation of apoptotic signaling, the involvement of additional pro- and anti-apoptotic regulators was not examined in the present study. Given the known link between ROS overproduction and caspase activation^37,38^, it is plausible that oxidative stress served as the upstream trigger for apoptosis, leading to germ cell loss and testicular degeneration.

In addition to oxidative and inflammatory damage, HANPs induced a marked upregulation of autophagy-related genes (*Beclin-1*, *LC3B*, *ULK1*, and *ATG9*). While basal autophagy supports spermatogenesis by removing damaged organelles, excessive activation—especially under oxidative stress—can contribute to cell death and tissue injury^11,39,40^. These findings suggest dysregulation of autophagy rather than definitive activation of a complete autophagic flux, which would require assessment of additional markers such as ATG5 or SQSTM1/p62. This aligns with previous observations that nanoparticles can trigger both apoptosis and autophagy through ROS-mediated mitochondrial dysfunction and lysosomal damage^41,42^.

Co-supplementation with *M. purpureus* RP markedly ameliorated HANP-induced reproductive toxicity in a dose-dependent manner, with the most pronounced protective effects observed at 40 mg/kg. RP alone was well tolerated and preserved normal testicular histology, semen quality, and hormonal balance. When co-administered with HANPs, RP significantly improved seminiferous tubule organization, restored interstitial tissue integrity, and normalized the epithelial lining with complete spermatogenic stages at the highest dose. These histological improvements paralleled functional recovery, as reflected by enhanced sperm motility, viability, and count, along with reduced sperm abnormalities. Importantly, these findings align with the growing body of evidence supporting green nanotechnology, an emerging field that emphasizes the use of biologically derived materials for the synthesis, functionalization, and modulation of nanoparticle behavior^43^. Within this context, RP represents a promising natural product capable of counteracting the adverse reproductive effects of nanoparticle exposure, although its precise molecular targets remain to be fully elucidated.

Moreover, molecular docking simulations showed that both monascorubramine and rubropunctamine exhibited higher binding affinities to the LC3B receptor than the control ligand GOL, primarily through hydrogen bonding with the key residue ARG37. Monascorubramine demonstrated the strongest and most stable interaction, indicated by its shorter bond length and higher negative energy score, suggesting a greater potential to modulate LC3B activity. These in silico findings provide supportive, but not definitive, evidence for a potential interaction with LC3B and should be interpreted as hypothesis-generating rather than confirmatory.

Mechanistically, RP co-treatment significantly reduced lipid peroxidation, replenished GSH, improved the GSH/GSSG ratio, and lowered oxidized GSSG levels, indicating strong antioxidant activity. These effects are consistent with earlier findings that *Monascus* extracts possess potent free radical-scavenging and antioxidant enzyme-inducing properties^16,17^. By alleviating oxidative stress, RP may indirectly influence downstream inflammatory, apoptotic, and autophagy-related responses rather than acting as a direct regulator of these pathways. In line with reports demonstrating that environmental endocrine disruptors such as bisphenol-S trigger oxidative stress-driven NF-kB activation and caspase-dependent apoptosis^44^, our results showed significant reductions in inflammatory and apoptotic markers, along with normalization of autophagy-related gene expression, in RP-treated HANP groups.

The protective effects of RP are further supported by studies reporting that *Monascus*-fermented products improve reproductive parameters by enhancing antioxidant enzyme activity, restoring testosterone levels, and reducing ROS generation and caspase activation^45^. In addition to its antioxidant capacity, RP exerts anti-inflammatory effects through NF-κB pathway inhibition and anti-apoptotic actions via caspase suppression. Together, these mechanisms likely contribute to, but do not fully account for, the protective phenotype observed in the present study.

The present study has several limitations that should be acknowledged. First, the experimental exposure was conducted using oral gavage in a rodent model, which may not fully reflect the complexity of human exposure routes, doses, or durations associated with HANP contact. Second, although key pathways related to oxidative stress, inflammation, apoptosis, and autophagy were investigated, additional signaling and regulatory markers were not assessed and would provide deeper mechanistic insight in future studies. Third, the molecular docking analysis offers supportive in silico evidence of potential interactions with LC3B but does not substitute for direct biochemical or functional validation at the protein level. Finally, while the findings demonstrate a clear protective effect of *Monascus purpureus* RPs under controlled experimental conditions, extrapolation to clinical or environmental settings should be approached with caution, and future studies are required to confirm efficacy under exposure scenarios more relevant to human health.

## Conclusion

In summary, HANP exposure induces male reproductive toxicity through interconnected mechanisms involving oxidative stress, inflammation, apoptosis, and dysregulated autophagy. Dietary supplementation with *M. purpureus* RP effectively mitigates these adverse effects in a dose-dependent manner, preserving testicular histology, sperm quality, and hormonal balance. These findings highlight the potential of RP as a natural, safe, and effective protective agent against nanoparticle-induced reproductive damage. Considering the growing use of HANPs in medical and industrial applications, such nutritional interventions could serve as a practical strategy to safeguard reproductive health and inform future translational studies.

## Data Availability

All data generated and analyzed in this study are included in this article.
